# Utilizing individual fish biomass and relative abundance models to map environmental niche associations of adult and juvenile targeted fishes

**DOI:** 10.1038/s41598-018-27774-7

**Published:** 2018-06-21

**Authors:** Ronen Galaiduk, Ben T. Radford, Euan S. Harvey

**Affiliations:** 1 Australian Institute of Marine Science, The University of Western Australia, 39 Fairway, Crawley, 6009 Australia; 2 School of Molecular and Life Sciences, Curtin University, Kent Street, Bentley, 6845 Australia; 3 The UWA Oceans Institute, The University of Western Australia, Fairway, Crawley, 6009 Australia; 4 School of Earth and Environment, The University of Western Australia, 35 Stirling Highway, Crawley, 6009 Australia

## Abstract

Many fishes undergo ontogenetic habitat shifts to meet their energy and resource needs as they grow. Habitat resource partitioning and patterns of habitat connectivity between conspecific fishes at different life-history stages is a significant knowledge gap. Species distribution models were used to examine patterns in the relative abundance, individual biomass estimates and environmental niche associations of different life stages of three iconic West Australian fishes. Continuous predictive maps describing the spatial distribution of abundance and individual biomass of the study species were created as well predictive hotspot maps that identify possible areas for aggregation of individuals of similar life stages of multiple species (i.e. spawning grounds, fisheries refugia or nursery areas). The models and maps indicate that processes driving the abundance patterns could be different from the body size associated demographic processes throughout an individual’s life cycle. Incorporating life-history in the spatially explicit management plans can ensure that critical habitat of the vulnerable stages (e.g. juvenile fish, spawning stock) is included within proposed protected areas and can enhance connectivity between various functional areas (e.g. nursery areas and adult populations) which, in turn, can improve the abundance of targeted species as well as other fish species relying on healthy ecosystem functioning.

## Introduction

The goals when designing marine reserves are usually the preservation of biodiversity and management of sustainable fisheries^1,2^. These goals are often constrained by economic considerations, which raise questions about where scarce conservation and fisheries management resources should be directed and which areas are most worthy of protection^3^. The decision about where to locate marine reserves to maximise biodiversity conservation and sustainable fisheries management outcomes is challenging, particularly when the conservation objectives are usually many and varied. Identifying key areas of the seascape that are crucial for multiple species, or for different life-history stages of same species (i.e. spawning grounds, fisheries refugia or nursery areas) can help to optimise the design and placement of reserves (e.g.,^4^) and may help to preserve critical spawning stock biomass of exploited species and result in lower losses and higher survival of vulnerable life stages^5^. Furthermore, protecting functional connectivity patterns between nursery areas and adult populations can enhance the abundance of target species as well as other fish species relying on healthy ecosystem functioning^1^.

Describing patterns of species-habitat associations has been the focus of many ecological and fisheries studies^6–9^. The quantity, type and quality of available habitat is known to influence the abundance, density and distribution patterns of many fishes^10^. Consequently, the identification of essential fish habitat has become a key goal for marine spatial management^11^. Species distribution models (SDMs) are a robust method for the rapid assessment of species-habitat associations at broad geographical scales^12,13^. In the last two decades, SDMs have become a common tool for investigating patterns in fish occurrence, abundance and density in relation to benthic marine habitats^14–17^. The results of predictive ecological modelling have helped to map and identify areas for spatial protection and to develop zoning and management plans for marine environments^4,12^.

It is common to base SDMs on occurrence^18,19^ and, more recently, abundance^20,21^ datasets. However, as underlying mechanisms that determine presence can be different to those that determine abundance^22^, examining other demographic processes such as species density or biomass estimates can enhance the potential benefits of using SDMs for spatial management applications. More specifically, since many demersal fish species undergo ontogenetic habitat associations as they grow^11,23^, incorporating the size structure or biomass measurements of individual fish could help to characterise the relationships between different life-history stages of individual conspecific fishes and the environment. The biomass of fish is often a major consideration in fisheries management, where in some cases major reproductive capacity could be invested in relatively few, old, large-size individuals that could produce exponentially more eggs than smaller size conspecifics^24,25^. The use of individual fish biomass in SDMs can enhance the spatially explicit management plans by ensuring critical habitat of the vulnerable life-history stages (e.g. juvenile fish, spawning stock) is included within proposed protected areas^14^.

In this study, we use SDMs to investigate the relationships between relative abundance, individual fish biomass estimates and benthic habitat structure at spatial scales relevant to informing regional marine spatial management.

The specific aims of this study were: (1) To model relative abundance/individual biomass and environmental niche associations of three iconic fish species (*Glaucosoma hebraicum, Choerodon rubescens* and *Chrysophrys auratus*) in Geographe Bay, Western Australia; (2) To compare and contrast the ecological performance of the developed models and the predictive maps of the continuous spatial distributions of the three species across the study area; (3) To create a predictive hotspot map as a single GIS layer to identify key areas for multiple species (i.e. nursery areas or spawning stock biomass hotspots), which can be informative for marine spatial management and planning.

## Results

### Model selection and variable contributions

**Figure 1:**
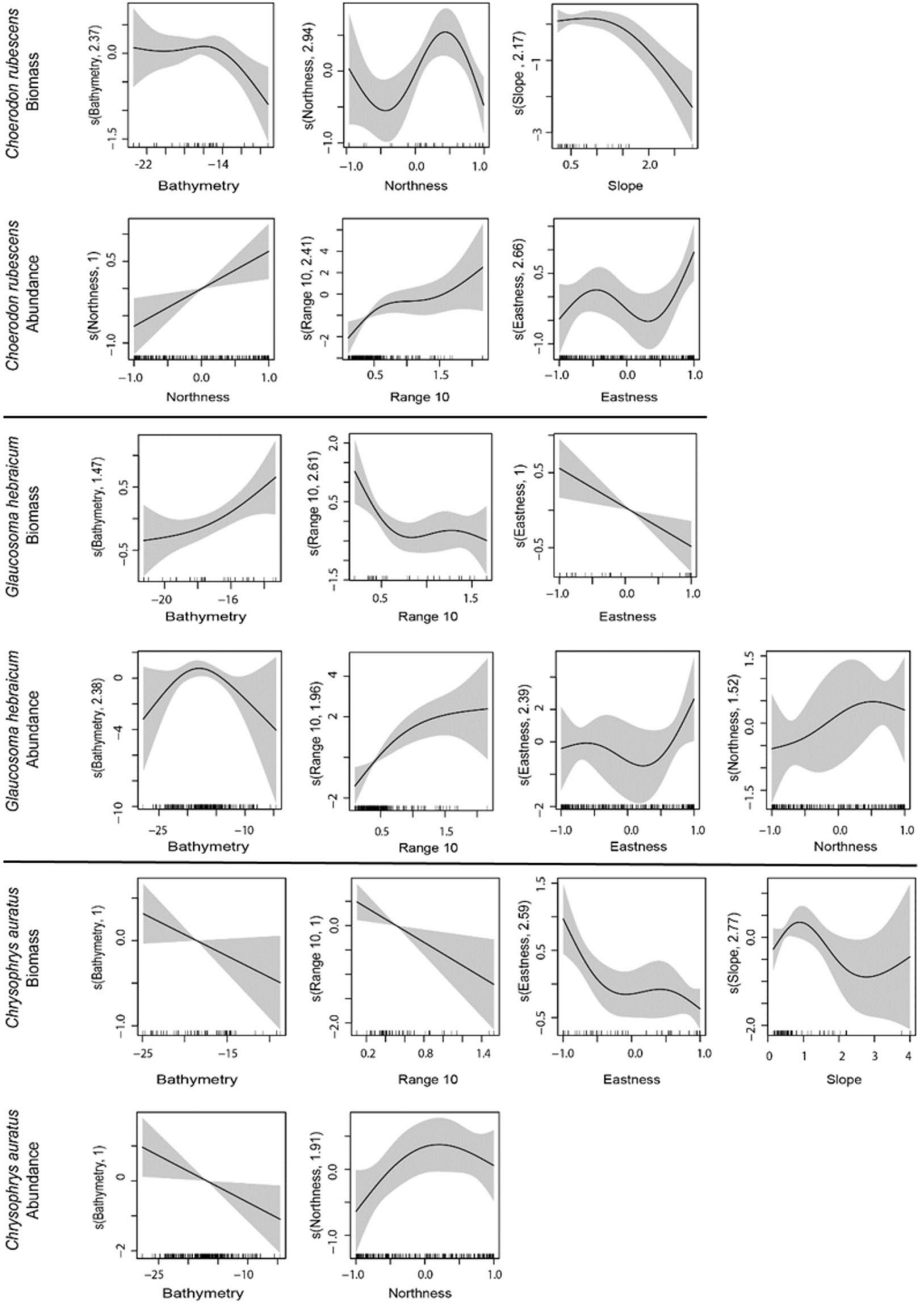
Smoother estimates (solid line) for the environmental predictors as obtained by generalised additive models for individual biomass and relative abundance of the three study fish species. The approximate 95% confidence envelopes are indicated (grey shading), marks along the x-axis are sampled data points. All explanatory variables were fitted with model smooths (knots) k = 4. Summary of the environmental predictors is provided in Table 1.

**Figure 2:**
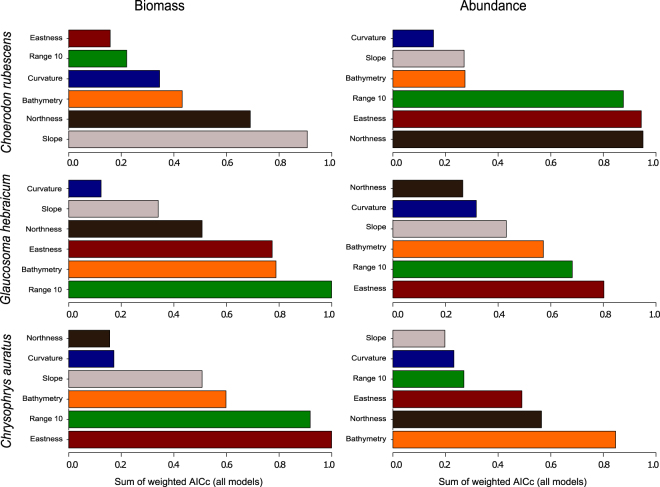
The relative importance of all environmental variables as indicated by the sum of weighted AICc for each variable across all fitted models for relative abundance and individual biomass of the three species.

Non-linear responses were frequently observed between the individual biomass or relative abundance of the study species and the explanatory environmental variables (Fig. 1). These non-linear responses provided strong support for using generalised additive models (GAMs) in studies of the relationships between demersal fish and their environment. The relative importance of the explanatory variables across all model fits was similar between the individual biomass and the relative abundance models for *Glaucosoma hebraicum* and *Chrysophrys auratus*, but differed for *Choerodon rubescens* (Fig. 2). The most commonly chosen variables across all model fits for all study species were depth (bathymetry), range (indication of structural complexity of the relief) and eastness (azimuthal direction of the reef slope) followed by northness and slope (Table 1, Fig. 2 and Supplementary Table S1 online for all candidate models).

**Table 1:** GAMs of best fit for predicting individual biomass and relative abundance distribution of the three study species. Best descriptor variables identified by (+).

	Species	Intercept	Bathymetry	Northness	Curvature	Range10	Eastness	Slope	Adjusted R^2^	df	AICc	∆AICc	Akaike weights	Normalized RMSE (%)
BIOMASS	*Choerodon rubescens*	6.45	+	+				+	0.61	9.48	501.0	0	0.40	23.5
	*Glaucosoma hebraicum*	6.84	+			+	+		0.45	7.08	552.9	0	0.38	27.9
	*Chrysophrys auratus*	7.14	+			+	+	+	0.33	9.36	1788.2	0	0.38	19.9
ABUNDANCE	*Choerodon rubescens*	−1.71		+		+	+		0.18	8.68	256.0	0	0.51	20
	*Glaucosoma hebraicum*	−2.38	+	+		+	+		0.28	11.58	220.8	0	0.34	14
	*Chrysophrys auratus*	−0.66	+	+					0.06	5.29	426.7	0	0.27	11.5

A full summary of candidate models (∆AICc < 2) is presented in Supplementary Table S1.

Depth was an important environmental variable for relative abundance and individual biomass of all modelled species except abundance of *C. rubescens* where it was assigned low importance (Fig. 2). Best fit models predicted lower biomass individuals and lower abundance in shallow areas for *G. hebraicum* and *C. auratus*, with the exception of the biomass of *G. hebraicum* where higher biomass individuals were predicted in shallow water (Fig. 1).

Range was an important variable for the relative abundance of *C. rubescens* and *G. hebraicum*, where a higher abundance of these species was predicted near reef edges (Fig. 1). Range was also important for individual biomass of *G. hebraicum* and *C. auratus*, where higher biomass individuals of these species were predicted for the areas of low complexity (Fig. 1). These results are particularly interesting for *G. hebraicum*, which exhibited reversed patterns in the abundance and individual biomass distributions. Similar patterns were observed for eastness variable. A higher abundance of *C. rubescens* and *G. hebraicum* and lower biomass individuals of *G. hebraicum* and *C. auratus* were predicted on the east-facing slopes (Fig. 1).

The explanatory power of the best models was notably higher for the individual biomass models (Table 1). However, the individual biomass models had slightly higher cross-validation errors (normalized RMSE). The best fit model developed for biomass of *C. rubescens* had the highest explanatory power across both the biomass and abundance datasets (adjusted R^2^ = 61%) and intermediate predictive error (normalized RMSE = 23.5%). While the predictive error for the model of the relative abundance of this species was highest with intermediate explanatory power (normalized RMSE = 20%; adjusted R^2^ = 18%; Table 1). Despite the fact that the best fit models developed for the individual biomass and relative abundance of *C. auratus* had the lowest associated predictive errors (normalized RMSE = 19.9 and 11.5% respectively), the explanatory power of these models was lowest across both the biomass and abundance datasets (adjusted R^2^ = 33% and adjusted R^2^ = 6% respectively; Table 1). The amount of cross-validation error could be associated with the sample size and the range of sampled biomass and abundance values. For example, the observed relative abundance values of *C. rubescens* and *C. auratus* ranged between 1–3 and 1–13, resulting in the highest and lowest error terms respectively. Similarly, the sample sizes for biomass of *C. rubescens* and *G. hebraicum* were 34 and 35 individuals respectively in the study area, with the range of observed biomass values almost twice larger for *G. hebraicum*, which evidentially resulted in the highest cross-validation error for the biomass models of this species. Sample size is known to have a major impact on model performance^26^.

### Model validation

The visual examination of residuals of models of best fit for the abundance of all modelled species identified the high frequency of negative residuals, which could be attributed to a large number of zeroes observed in these datasets. In the exploratory stages of our analysis, we examined the possibility of applying the zero-inflated Poisson GAMs to the abundance dataset. However, the zero-inflated models with Poisson error distribution did not resolve the negative skewness in the residuals and produced higher cross-validation errors, thus supporting our choice of the modelling approach. At this stage, only one package compatible with R statistical software is still under development that will allow fitting zero-inflated GAMs with negative binomial error distribution that could provide a potential solution to the negatively skewed residuals^27^.

**Figure 3:**
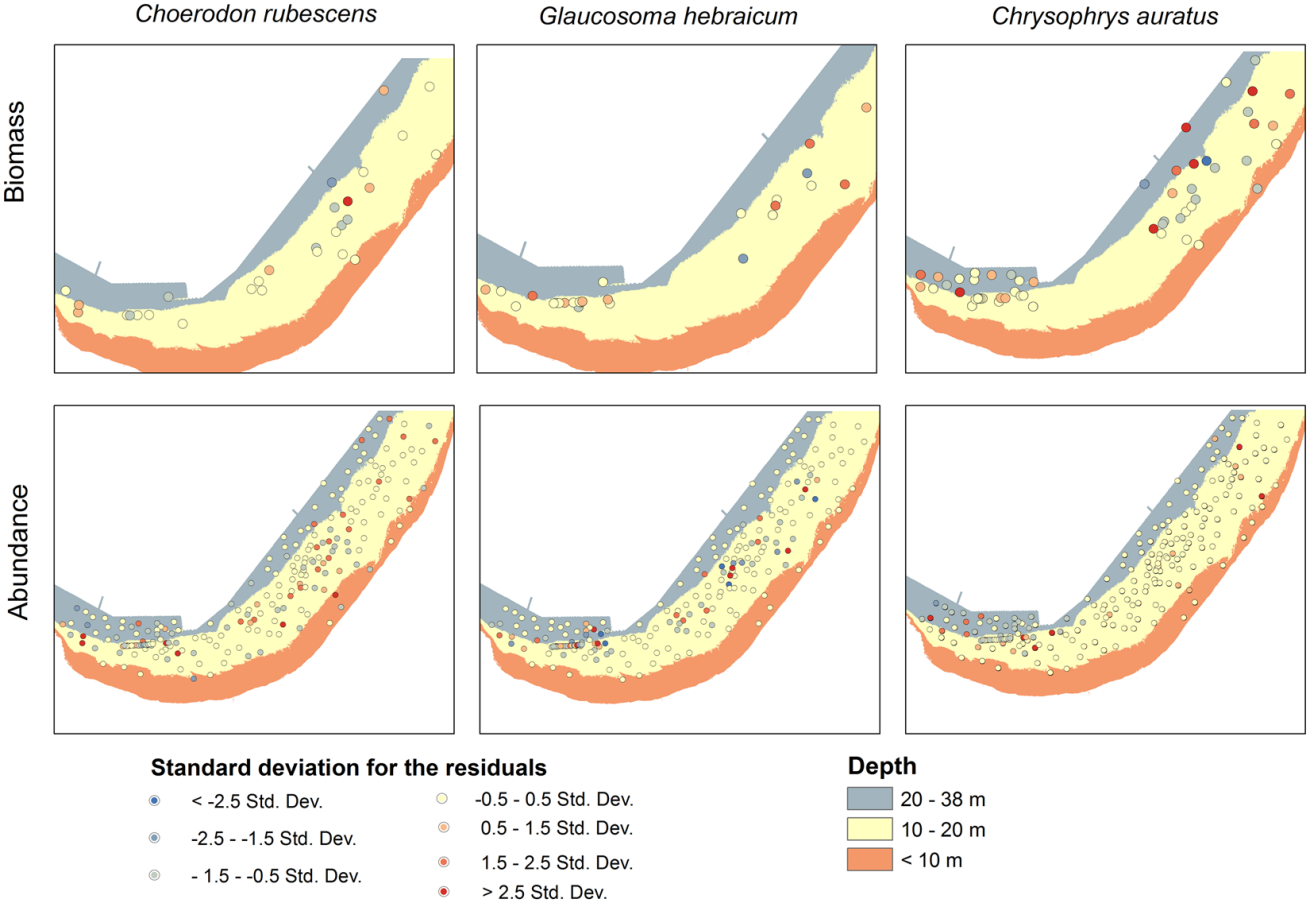
Distribution of the local standardised residuals from the Geographically Weighted Regression analysis for the relative abundance and individual biomass models.

There was a small amount of spatial clustering of high residuals in the north-eastern part of the bay in GWR model fit for individual biomass of *Chrysophrys auratus* (Fig. 3). In addition, there was some degree of spatial clustering of the high and low residuals in GWR models fitted for the relative abundance of all study species. However, the Moran’s I analysis on the standardised residuals of all GWR models did not indicate spatial correlation in model residuals (all Z scores represented the expected outcome and all *P* > 0.05) suggesting that our models were well parametrised with respect to geomorphic/spatial variables. Thus, we conclude that the observed high/low residual patterns are due to local habitat characteristics, such as the extent of canopy cover or occurrence of sessile invertebrates, that could drive behaviour responses of *C. auratus* and are known to produce patterns in model residuals^28–30^.

### Spatial predictions

**Figure 4:**
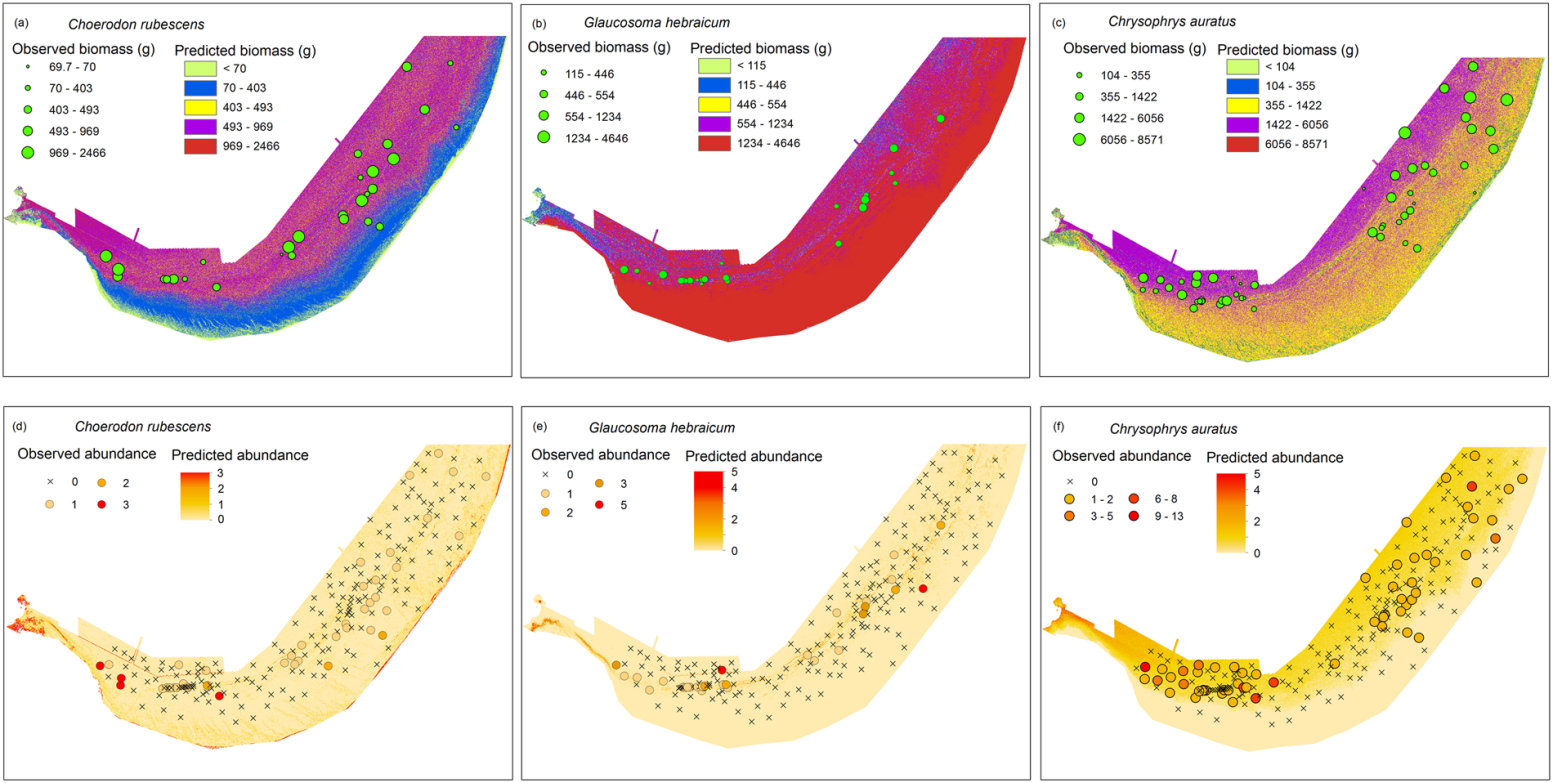
Predicted maps of continuous distributions of the three species across Geographe Bay for the individual biomass and relative abundance as defined by the GAMs of best fit for individual study species. Observed individual biomass and relative abundance estimates as well plotted.

The predictions from the models of best fit provided a continuous representation of environmental niche suitability for individual biomass and relative abundance distributions of the study species across the entire Geographe Bay (Fig. 4). The small biomass individuals of *Choerodon rubescens* were predicted to be associated with shallow, protected south or east facing reef edges, whereas the large biomass individuals were predicted to be found in deeper, flat areas of the bay (Fig. 4a). The high abundance of this species was predicted for exposed reef edges particularly in the western part of the bay (Fig. 4d).

The large biomass individuals of *Glaucosoma hebraicum* were predicted to be found in shallow, low relief westward sloping areas of the bay. In contrast, small biomass individuals of this species were predicted to be associated with deeper protected near reef areas of the bay (Fig. 4b). The high abundance of *G. hebraicum* was predicted for the north or east facing near reef areas at intermediate depths (Fig. 4e).

The small biomass individuals of *Chrysophrys auratus* were predicted to be associated with shallow, east facing high relief reef areas of the bay, whereas the large biomass individuals of this species were predicted to be found in deep flat areas in the west part of the bay (Fig. 4c). The high abundance of this species was predicted in the deep and exposed western part of the bay (Fig. 4f).

**Figure 5:**
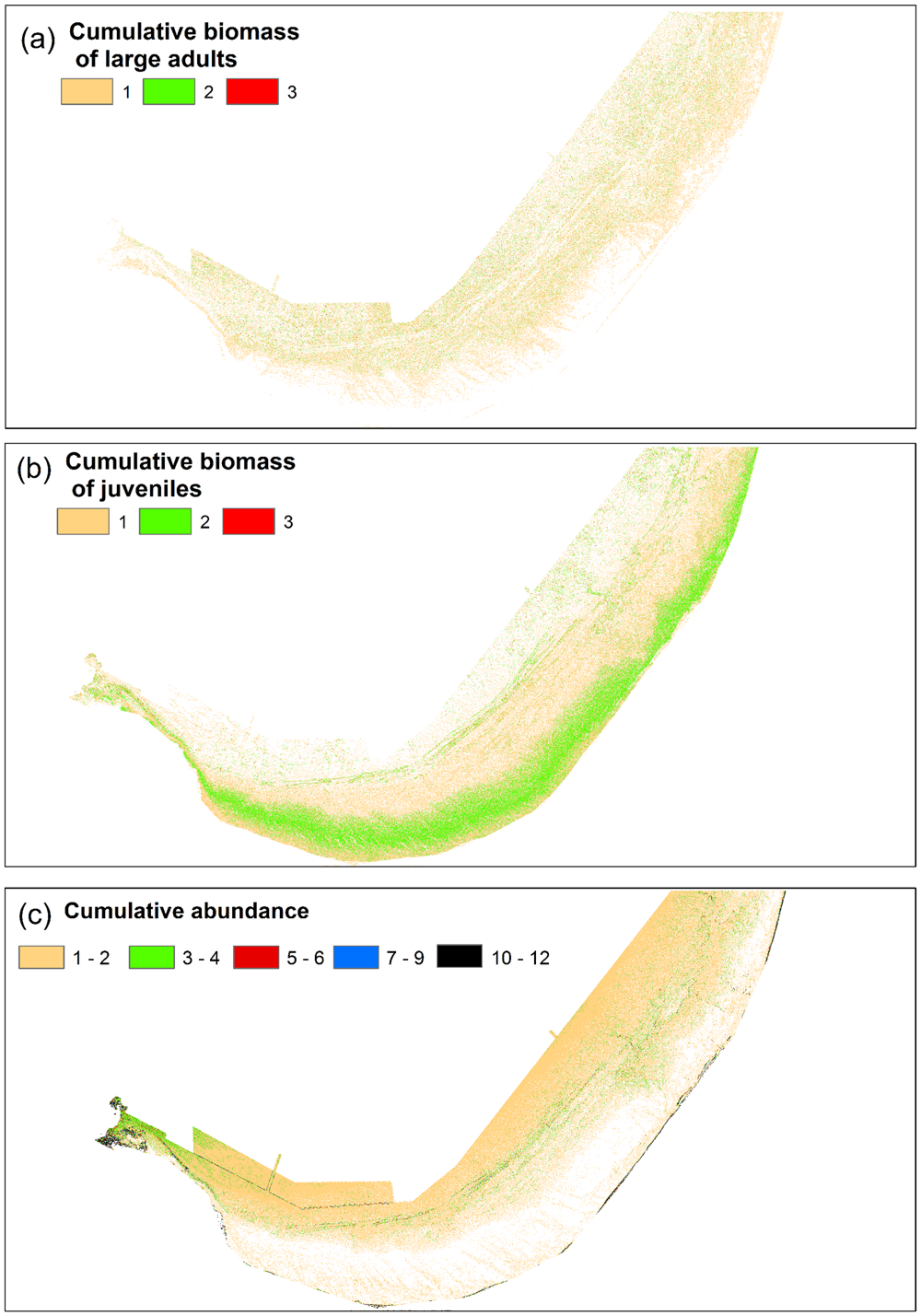
Hotspots map for cumulative biomass of large sexually mature adults (**a**), juveniles (**b**) and cumulative predicted abundance (**c**) of the three study species. Rasters reclassified based on known biomass estimates of the study species.

Cumulative predictive maps of relative abundance and individual biomass of small/juvenile and large/mature adults of all three study species identified shallow coastal areas of the bay as being a hotspot for aggregation of small fish biomass (Fig. 5b). In addition, small local pockets of aggregations of small and adult fish biomass were identified from the cumulative maps across the bay. However, no additional distinctive hotspots for the study species could be assumed from the cumulative maps of individual biomass (Fig. 5a,b). The reef ridge areas that span across most of the bay was predicted to be characterised by a high abundance of individuals of the study species with higher cumulative predicted abundance in the western part of the bay (Fig. 5c). The predicted high abundance in the western part of the bay could be further validated with a ‘ground-truth’ data collection for this part of the bay which was not covered in this study.

## Discussion

This study successfully combined abundance estimates and individual fish size measurements with high-resolution hydroacoustic surveys in the spatial modelling domain to produce predictive maps that can inform management efforts of three highly sought after fish species. Modelling spatially explicit patterns of abundance and biomass can help to underpin regional population dynamics, enhance associated populations and inform spatial conservation approaches designed to protect biodiversity^14^. Our findings highlight the potential vulnerability of both the juvenile and the sexually mature adults of the three species which rely on the near shore areas and this important outcome would have been missed if only the abundance patterns of these species had been considered. Therefore, we suggest using biomass and abundance models to complement each other.

We found that depth, structural complexity of habitat and direction of reef slope were the most common predictors of the observed patterns in relative abundance and individual biomass distribution of the three key indicator species. However, the relative importance of all explanatory variables varied between species for relative abundance and individual biomass models suggesting that a different hierarchy of environmental processes dictates patterns in species-specific abundance and biomass distributions. Depth and structural complexity are also indicative of key processes that relate to resilience in other systems, such as regime shifts on coral reefs^31^ and they should be considered as part of selection criteria for spatial planning of marine reserves.

Small-scale habitat characteristics have previously been documented to influence the abundance and diversity of reef fishes^32,33^ and to drive species-specific response to the environment^34^. The western part of the bay was predicted to be a hotspot for the cumulative abundance of the three modelled species. In addition, higher abundances of West Australian dhufish (*Glaucosoma hebraicum*) were predicted along the reef ridges across the bay. The observed high abundance gradient of the three species in the ocean-ward part of the bay could be driven by the large-scale population dynamics of these species. The pre-settlers of the three species were recorded to utilise major regional oceanic currents such as south-ward flowing Leeuwin Current or north-ward flowing Capes Current for enhanced larval transport from the source of populations further along the coast^9,35^. Therefore, higher abundances of the three species could be expected in the areas close to the source of transport, gradually declining in the inner part of the bay. Similar patterns were documented in the case of the Mediterranean wrasse, with a greater abundance of this species observed in the areas that were closer to the source of population^36^. High structural complexity and higher prey availability on reef habitats could be additional factors in explaining high abundance of *G. hebraicum* near the reef ridges. This carnivorous fish is known to favour reef habitats at various life-stages^37^.

The predicted distribution and the extent of ecological niches across the bay were similar for individual biomass of the Baldchin groper (*Choerodon rubescens*) and Australasian snapper (*Chrysophrys auratus*) identifying shallow coastal areas with high structural complexity as the most suitable hotspot area for juveniles of these species. Deeper areas of the bay with high complexity relief were also found to be good predictors of biomass distribution of juvenile *G. hebraicum*. In contrast, mature adults of this species were predicted to be associated with shallow coastal waters. However, the hotspot maps of the cumulative biomass of large, sexually mature fish did not indicate any parts of the bay as being crucial for this stage of the species’ life history. A difference in mobility and the size of home ranges between adult and juvenile fishes are most probably the reasons that the hotspots for adult individuals were not identified in our study. While adult fishes are typically more mobile with relatively large home ranges, small-size fish have smaller home ranges and are less likely to move as far as larger bodied conspecifics^38^. In addition, a variety of juvenile fish are known to exhibit relatively high site fidelity with structurally complex habitats using them as their nursery areas^39^ or predation refugia^40^, which may have helped to identify the environmental niche requirements of juvenile fish more accurately.

Incorporating size structure can inform the design of conservation approaches such as protected areas and also be used to estimate larval production and the contribution of protected populations to the replenishment of populations within and outside of protected areas^14^. In many cases, abundance and biomass patterns could often produce very different curves for the same species^41^. There is often a shift associated with the establishment of marine reserves, where fished sites are characterised by higher abundance than biomass and protected sites by higher biomass of large-bodied species than the relative abundance of species^42^. While species’ abundance is clearly an important measure, individual biomass estimates could be more relevant for explaining patterns of resource use or niche partitioning among conspecifics than abundance models^43^.

As a fisheries management tool, pairing video observations and measurements with remotely sensed (hydroacoustic or LIDAR) benthic habitat data with species distribution models has tremendous potential for understanding fine-scale species-environment relationships of demersal fish. In addition, mapping key areas of a seascape that are crucial for different life-history stages of the same species or multiple species may benefit actively fished species, particularly those species that exhibit high site fidelity and relatively localised movement patterns. A novel implementation of analytical tools, such as the Geographically Weighted Regression for fisheries management, can simultaneously confirm model parametrisation quality and identify clustering patterns in the residuals (hot/cold spots, *sensu*^28^) which could be evidence of fish aggregation pockets and can highlight areas of further exploration and/or management intervention. Implementing management actions in the hotspot areas that provide protection from disturbance, such as bycatch or undersize fishing, may result in lower losses and higher survival of vulnerable life stages of targeted and non-targeted species, which in turn can preserve critical spawning stock biomass of exploited species, enhance fishery yields outside the protected hotspot areas and promote overall healthy functioning of the ecosystem^5^.

Nursery areas contribute to adult population patterns^44^. Enhancing the ability to monitor juvenile recruitment variability in areas of critical juvenile habitat would, for some species, allow predictions of future strength of cohorts to be made before they enter the fishery^7^. By creating temporary closures of adult breeding grounds during spawning season, it is possible to enhance the reproductive dynamics of the entire population of the target species. For example, longer spawning season and a larger amount of eggs per batch are documented for large mature females of *G. hebraicum* in comparison to the smaller mature females making them extremely vulnerable to fishing during the spawning period^45^. Protecting the crucial areas of seascape for large sexually mature females could enhance the abundance and biomass of depleted stocks^46,47^, which in turn can benefit other fish species relying on healthy ecosystem functioning e.g.,^1^. Furthermore, the hotspot areas may preserve critical spawning stock biomass of exploited stocks more effectively than size limits and catch quotas for some species by preserving natural size distributions and densities^5^. Our study provides a novel approach that can be incorporated into efforts to address this knowledge gap for a wide variety of species. The hotspot maps can optimise limited management resources by identifying entire areas that may not require future in-depth surveys. Following *in situ* evaluation of the predicted hotspots, these areas should be considered in zoning schemes and become priority areas for marine spatial monitoring and management^48^. Such an approach could be extremely useful for spatial management when mapping distribution patterns in fish diversity, and for understanding of population dynamics of endangered species.

## Methods

### Study area

**Figure 6:**
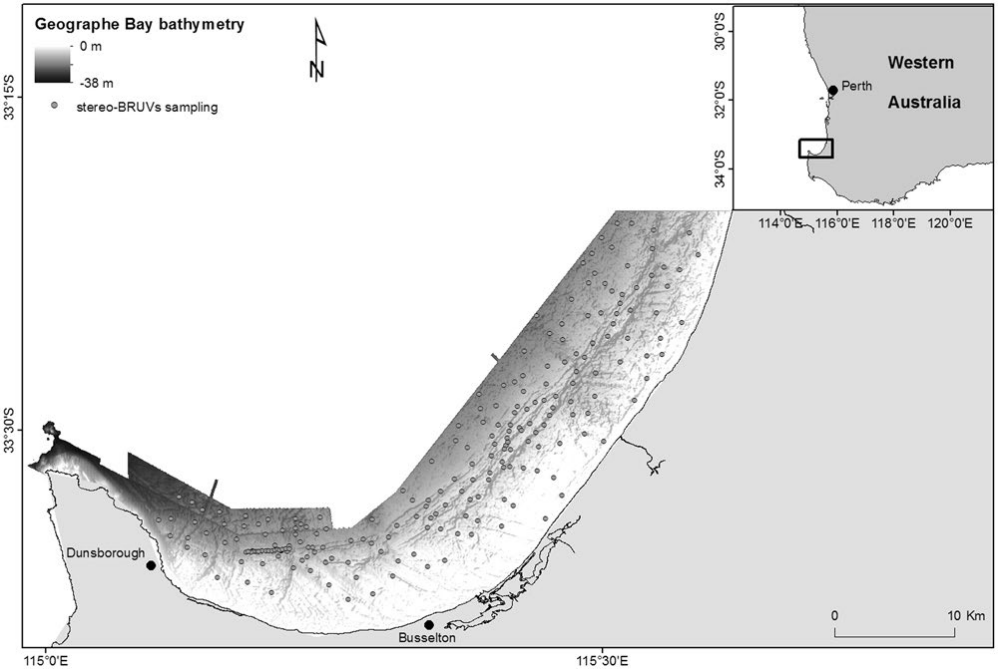
Bathymetry of Geographe Bay with grey dots indicating the stereo-video deployment sites. Inset: The location of the Geographe bay study area on the south-west coast of Western Australia.

Geographe Bay is a ~100 km wide, relatively shallow, north-facing embayment with seagrass cover that can at times exceed 60%^49^. The bay is located in southwestern Australia, approximately 220 km south of Perth (Fig. 6). It is part of Ngari Capes Marine Park with approx. 3500 ha (<4% total bay area) zoned as no-take marine sanctuary^50^. The majority of the seafloor is covered by unconsolidated sediments that have been deposited over older clay layers. There is also a series of discontinuous limestone ridges, dominated by canopy-forming brown macroalgae, that run parallel to the coast^51,52^.

### Fish abundance and biomass data

This research was conducted in accordance with all relevant guidelines and regulations following permits AEC_2014_21 and SF009757 issued by the Curtin Animal Ethics Committee and WA Department of Parks and Wildlife respectively. We collected data on the patterns of relative abundance and individual biomass of three iconic West Australian fishes: dhufish (*Glaucosoma hebraicum*), baldchin groper (*Choerodon rubescens*) and Australasian snapper (*Chrysophrys auratus*). These are indicator species for fisheries management in Western Australia and account for the majority of the total nearshore and estuarine catch by commercial and recreational fishers^53,54^. Stock assessments classify these species as being overfished along the central west coast region of Western Australia^45,55^ despite the implementation of common fisheries management strategies, such as bag and size limits, licensing and quotas.

The relative abundance and biomass of these three target fish species were surveyed between the 9th-17th December 2014 using baited remote underwater stereo-video systems (hereafter stereo-BRUVs). This method of data collection is thought to be optimal for sampling large, mobile, carnivorous fish that are low in abundance^7,56^. Each stereo-BRUV system comprised two wide-angle Sony CX12 high-definition video cameras that had been baited with approximately 1000 g of crushed pilchards (*Sardinops sagax*), and lowered to the bottom for a 60 minute soak time. The 217 video recordings from these deployments were analysed using the software EventMeasure (SeaGIS Pty Ltd). For sample unit standardisation purposes and to ensure high measurement accuracy and precision we only included fish within 7 m in front and 3 m into the water column above the system. Additional information on design, calibration^57,58^ and use of the stereo-BRUVs is presented in detail in the literature (e.g.,^59,60^ and references therein). To ensure that sampling replication was appropriate, random stereo-BRUVs deployments were spatially stratified according to the size of the study area, habitat availability and depth: random points for sampling were allocated to adequately cover the depth gradient in the bay, although major substrate types (e.g. reef ridge) were particularly targeted based on the skipper’s local knowledge of the study area^61^. In addition, distance controls were used in the planning stage to avoid bait plume overlap and reduce the likelihood of fish moving between stereo-BRUVs, with each pair of stereo systems at least 400 m apart from each other on the day of deployment. The relative abundance of study species was estimated using MaxN^62–64^. This measure is considered to be conservative for estimating fish abundance and avoiding repetitive counts of individual fish in 1 hour long recordings^60^. The fork length of individuals at the MaxN of each species was measured for each stereo-BRUVs deployment with the EventMeasure software (www.seagis.com.au) with precision constraints set to a 10% cut off, which is achievable using stereo-BRUVs^57,65^. The biomass estimates for individual fish observed in the video recordings were obtained with previously estimated length-weight relationships^54^ and references therein. For *Glaucosoma hebraicum*, the length-weight relationships are different for males and females. We were unable to sex the individual fish in the video recordings, therefore the biomass estimates were averaged for male and female individuals of this species.

### Environmental variables

The bathymetric data was extracted from a mosaic of LiDAR and multibeam surveys collected by Fugro Corporation Pty Ltd gridded to a cell size of 4*4 m. The LiDAR hydrographic survey was performed between April and May 2009 on behalf of the Department of Planning as a part of a national coastal vulnerability assessment. The LiDAR area extended seaward from the coastal waterline to the 20 m marine nautical navigation chart contour and constituted the majority of bathymetric data (for details on LiDAR collection and processing see www.planning.wa.gov.au, accessed May 2016). In addition to the LiDAR, a small area of deeper water was surveyed during March-April 2006 using Reson 8101 multibeam in the north-west part of the study area as part of the Marine Futures biodiversity surveys (see^66^ and www.matrix-prod.its.uwa.edu.au/marinefutures; accessed May 2016 for further details). In addition to the bathymetric data, we derived five additional environmental variables from the mosaiced survey grids that describe the structure and complexity of the seafloor and were previously shown to influence the distribution of fish using the Spatial Analyst toolkit in ArcGIS 10.2.2^16,17^ (Table 2).

**Table 2:** Description of the environmental predictors extracted from the hydroacoustic surveys used to fit GAMs.

Environmental Predictor	Description
Bathymetry	Elevation in metres relative to the Australian Height Datum.
Eastness	Trigonometric transformation of a circular azimuthal direction of the slope (*sin*(aspect)). Values close to 1 represent east-facing slope, close to −1 if the aspect is westward.
Northness	Trigonometric transformation of a circular azimuthal direction of the slope (*cos*(aspect)). Values close to 1 represent north-facing slope, close to −1 if the aspect is southward.
Slope	First derivative of elevation. Average change in elevation, steepness of the terrain, % rise.
Range 10	Maximum minus the minimum elevation in the local neighbourhood (coarse scale local relief). Calculated at window size of 10*10 cells, which equates to ground area of 1600 m^2^.
Curvature	Combined index of profile (parallel to the slope) and plan (perpendicular to the slope) curvature relative to the analysis window.

### Species distribution modelling

To infer the effect of habitat complexity on the relative abundance and individual biomass of three fish taxa we applied GAMs developed for individual study species and the full-subsets Information Theory approach^67^. This approach is based on assumption that no statistical model exactly represent reality but the relative proximity to absolute reality could still be quantified amongst a set of candidate models to afford valuable insight achieved by considering the relative performance of some or even all of the candidate models^29,68^. The combination of GAMs, which is the most common and well-developed method for modelling fish-habitat relationships^12,48,69^, and the Information Theory provided an unconstrained approach for fitting ecological responses to the predictor variable^29,70^. The initial data exploration followed procedures outlined in^71,72^, examining potential outliers, homogeneity and co-linearity of covariates for subsets of data for individual fish species. There were large slope values observed in the exploratory stage. However, we decided to keep these potential outliers, as they represent the true nature of the benthos of the bay which is mainly characterised by low relief seascape with occasional reef ridges.

The GAMs for relative abundance estimates, which were characterised by a large proportion of zeroes, were fitted with negative binomial error distribution and a logarithmic link function. The decision to use the negative binomial error distribution was made after comparing the observed frequency distribution of relative abundance values to theoretical density curves from a negative binomial and a Poisson distributions (which are the most common types of statistical distributions for analysing count data^73^) for similar mean and dispersion parameters^29,74^. The frequency distribution for the observed relative abundance values for all focal species best resembled the distribution of theoretical values from the negative binomial density curves. The individual biomass GAMs were fitted with gamma error distribution and logarithmic link function, which is a suitable statistical distribution for analysis of a continuous positive response variable^29,75^.

Due to the amount of data available for model fitting, and to produce conservative models^76^, the maximum number of explanatory variables across all fitted models was limited to four, as well as the maximum number of knots which was restricted to k = 4. To minimise the probability of model overfitting, the model fits for all possible combinations of variables were compared using the Akaike Information Criterion corrected (AICc), which is a recommended criterion for finite sample size^67^. In addition, to rank the fitted models we computed the Akaike weights^77^ to examine the weight of likelihood in favour of a model being the best in the given set of models. To explore the relative importance of each predictor variable, we summed the weighted AICc values across all models in the set where the individual predictors occur. The larger the sum of the Akaike weights, the more important the variable is in relation to all the other variables^67^. When number of candidate models tied for best for data analysis (arithmetic difference between a model AICc and the minimum AICc for all models, denoted ∆AICc < 2), the model of best fit was selected based on having the highest Akaike weight ranking for likelihood of evidence across all possible models *sensu*^67^. Response curves were visually inspected for ecological realism^78^. All models were fitted in R version 3.2.0^79^.

### Model validation

Models of best fit for individual biomass and relative abundance estimates were cross-validated using 5-Fold cross validation 50 times^80^. The premise of this cross-validation method is splitting the dataset into five roughly equal parts and repeatedly fitting a model to four parts of the data while using the remaining part of the data for model testing^81^. We then calculated the normalized root mean square error (normalized RMSE) to examine the average magnitude of the predictive errors of all generated submodels^82,83^. Plots of model residuals were visually investigated for patterns following the procedures outlined in^29,72^.

To investigate any residual spatial patterns not accounted for with the relationships between the observed biomass/abundance and values predicted by the models of best fit, we fitted geographically weighted regression (GWR) and examined the spatial patterns in the distribution of the local standardized residuals^84^. The GWR allows for nonstationarity in the relationships between the dependent (observed biomass/abundance) and the explanatory (predicted biomass/abundance) variables and is a useful explanatory technique for interpretation based on spatial context and known characteristics of the study area^85^. It also has the potential to identify the scale of missing model variables and identify other spatial patterns in data not driven by input variables (for example behaviours such as spawning and feeding aggregations e.g.,^28^).

### Spatial prediction of species’ biomass and abundance

Once the best fit models were validated, the constrained individual biomass and relative abundance estimates of three fish species were predicted separately on 4 m grids using R and these predictions were plotted in ArcMap 10.2.2. To identify hotspot areas where large fertile adults or small juvenile fish of the three species tended to aggregate, the continuous predictive biomass rasters were reclassified into these two categories according to the known biology and the life history of individual study species. The cutoff points for the reclassification process were based on the individual biomass values (which act as a proxy to individual’s fecundity) summarized for the three species in^54^. The reclassified values were plotted again to map the hotspot areas where juvenile or mature adult fish of the modelled species aggregate. For example, a hotspot for juvenile/mature fish will have a maximum score of 3, corresponding to juvenile/mature individuals of the three modelled fish species that can potentially associate with that particular area. In addition, the predictive fish abundance rasters were summed for all study species to identify areas of Geographe Bay associated with a high cumulative abundance of individuals of the modelled species.

### Data Availability

The datasets analysed during the current study are available from the corresponding author on reasonable request.

## Electronic supplementary material

Supplementary table S1
