# Supplementary material for: Utilizing individual fish biomass and relative abundance models to map environmental niche associations of adult and juvenile targeted fishes
Source: Sci Rep. 2018 Jun 21;8:9457. doi: 10.1038/s41598-018-27774-7 (PMC6013477; doi:10.1038/s41598-018-27774-7)
Supplement: Supplementary file 1 — Supplementary table S1 [file 41598_2018_27774_MOESM1_ESM.pdf]

Utilizing individual fish biomass and relative abundance models to map environmental niche associations of adult and juvenile targeted fishes

Ronen Galaiduk[1,2,*], Ben T. Radford[1,3,4], Euan S. Harvey[2]

[1]Australian Institute of Marine Science, The University of Western Australia, 39 Fairway, Crawley 6009, Australia

[2]School of Molecular and Life Sciences, Curtin University, Kent Street, Bentley 6845, Australia

[3]The UWA Oceans Institute, The University of Western Australia, Fairway, Crawley 6009, Australia

[4]School of Earth and Environment, The University of Western Australia, 35 Stirling Highway, Crawley 6009, Australia

[*]email: r.galaiduk@aims.gov.au

Supplementary table S1. Best descriptor variable (+) and the summary of candidate models (ΔAICc < 2) for predicting environmental niche associations for individual biomass and relative abundance distribution of the three study species. GAMs of best fit identified by ΔAICc = 0 and highest Akaike weights for evidence support.

	Species	Intercept	Bathymetry	Northness	Curvature	Range10	Eastness	Slope	Adjusted R^2	df	AICc	ΔAICc	Akaike weights	Normalized RMSE (%)
BIOMASS	*Choerodon rubescens*	6.45	+	+				+	0.61	9.48	501.0	0	0.40	23.5
	Choerodon rubescens	6.48		+				+	0.48	6.98	501.3	0.29	0.34	
	Choerodon rubescens	6.47		+	+			+	0.52	7.95	501.8	0.82	0.26	
	Glaucosoma hebraicum	6.84	+			+	+		0.45	7.08	552.9	0	0.38	27.9
	Glaucosoma hebraicum	6.82			+	+	+	+	0.5	8.34	553.6	0.65	0.27	
	Glaucosoma hebraicum	6.83	+			+	+	+	0.48	7.99	554.2	1.28	0.20	
	Glaucosoma hebraicum	6.84	+		+	+	+		0.47	7.95	554.8	1.83	0.15	
	Chrysophrys auratus	7.14	+			+	+	+	0.33	9.36	1788.2	0	0.38	19.9
	Chrysophrys auratus	7.15	+			+	+		0.3	8.28	1789.0	0.79	0.26	
	Chrysophrys auratus	7.16				+	+		0.29	7.29	1789.6	1.35	0.19	
	Chrysophrys auratus	7.16				+	+	+	0.28	6.78	1789.9	1.69	0.16	

ABUNDANCE	*Choerodon rubescens*	-1.71		+		+	+		0.18	8.68	256.0	0	0.51	20
	Choerodon rubescens	-1.74	+	+		+	+		0.2	11.06	257.2	1.18	0.28	
	Choerodon rubescens	-1.70		+		+	+	+	0.18	9.30	257.8	1.82	0.21	
	Glaucosoma hebraicum	-2.38	+	+		+	+		0.28	11.58	220.8	0	0.34	14
	Glaucosoma hebraicum	-2.28	+			+	+		0.26	9.88	221.2	0.42	0.27	
	Glaucosoma hebraicum	-2.35	+			+	+	+	0.27	11.05	221.2	0.45	0.27	
	Glaucosoma hebraicum	-2.29	+	+		+		+	0.26	10.94	222.8	1.96	0.13	
	Chrysophrys auratus	-0.66	+	+					0.06	5.29	426.7	0	0.27	11.5
	Chrysophrys auratus	-0.69	+	+			+		0.08	6.64	427.1	0.39	0.22	
	Chrysophrys auratus	-0.66	+				+		0.07	5.68	427.1	0.39	0.22	
	Chrysophrys auratus	-0.60	+						0.04	3.00	427.5	0.76	0.18	
	Chrysophrys auratus	-0.69	+	+		+			0.08	7.54	428.6	1.83	0.11	
